# Microbial engineering for the production of isobutanol: current status and future directions

**DOI:** 10.1080/21655979.2021.1978189

**Published:** 2021-12-19

**Authors:** Nair M Lakshmi, Parameswaran Binod, Raveendran Sindhu, Mukesh Kumar Awasthi, Ashok Pandey

**Affiliations:** a Microbial Processes and Technology Division, CSIR-National Institute for Interdisciplinary Science and Technology (Csir-niist), Thiruvananthapuram Kerala, India; b Academy of Scientific and Innovative Research (Acsir), Ghaziabad, Uttar Pradesh India; c College of Natural Resources and Environment, North West a & F University, Yangling, Shaanxi China; d Centre for Innovation and Translational Research CSIR-Indian Institute of Toxicology Research (Csir-iitr), Lucknow India; e Centre for Energy and Environmental Sustainability, Lucknow Uttar Pradesh, India

**Keywords:** Isobutanol, consolidated bioprocessing, cell free system, downstream process

## Abstract

Fermentation-derived alcohols have gained much attention as an alternate fuel due to its minimal effects on atmosphere. Besides its application as biofuel it is also used as raw material for coating resins, deicing fluid, additives in polishes, etc. Among the liquid alcohol type of fuels, isobutanol has more advantage than ethanol. Isobutanol production is reported in native yeast strains, but the production titer is very low which is about 200 mg/L. In order to improve the production, several genetic and metabolic engineering approaches have been carried out. Genetically engineered organism has been reported to produce maximum of 50 g/L of isobutanol which is far more than the native strain without any modification. In bacteria mostly last two steps in Ehrlich pathway, catalyzed by enzymes ketoisovalerate decarboxylase and alcohol dehydrogenase, are heterologously expressed to improve the production. Native *Saccharomyces cerevisiae* can produce isobutanol in negligible amount since it possesses the pathway for its production through valine degradation pathway. Further modifications in the existing pathways made the improvement in isobutanol production in many microbial strains. Fermentation using cost-effective lignocellulosic biomass and an efficient downstream process can yield isobutanol in environment friendly and sustainable manner. The present review describes the various genetic and metabolic engineering practices adopted to improve the isobutanol production in microbial strains and its downstream processing.

## 1. Introduction

**Figure 1.**
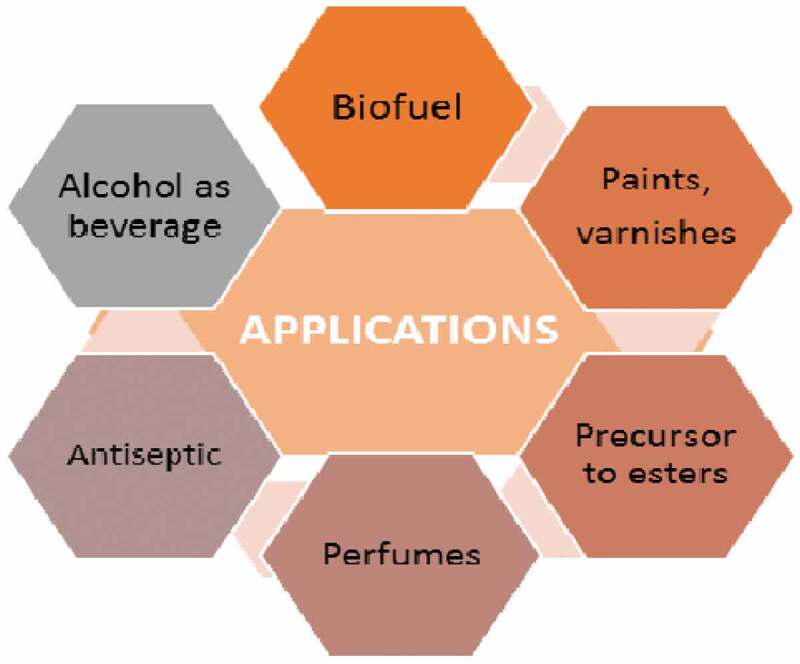
Applications of isobutanol

Isobutanol is a four-carbon aliphatic alcohol. The major application of isobutanol as biofuel has urged importance in past few years. Various applications of isobutanol are depicted in Figure 1. Among butanols, isobutanol is the least toxic one with LD_50_ of 2460 mg/kg. Isobutanol is also further converted into various other useful chemicals such as isobutyl acrylate, isobutyl acetate [1]. Bioethanol was used as a biofuel due to the present scenario where we focus more on environmental issues like global warming and water flooding. Nowadays this bioethanol is largely being replaced by biobutanol. Advantages of biobutanol over bioethanol include lower vapor pressure, could be blended at a higher ratio, less corrosive and lower oxygen content [2].

Isobutanol can be produced chemically as well as biologically. Petrochemical method of isobutanol production includes mainly two processes – the oxo synthesis and the Reppe synthesis. Oxo synthesis includes hydroformylation of propene to yield aldehyde which is further hydrogenated using catalysts like Co, Rh, or Ru to form corresponding alcohol n-butanol and isobutanol. In Reppe synthesis, propene, carbon monoxide, and water react in the presence of catalyst tertiary ammonium salt or polynuclear iron carbonyl hydrides at high temperature and pressure to form n-butanol and isobutanol [3]. Besides these methods various other chemical methods are also reported which include Guerbet reaction in which methanol/n-propanol condensation by using copper based catalytic system [4]. Another method includes glycerin and methanol fed to yield isobutanol [5].

In recent years due to the environment concerns like greenhouse gas effect and global warming biological methods are mostly preferred. Gevo and Butamax advanced biofuels are the two companies producing bio-based isobutanol. Various patents-related isobutanol production is depicted in Table 1. Isobutanol is preferred over bioethanol and n butanol due to its high octane number, less corrosive and is more compatible with existing gasoline.

**Table 1.** Patents related to isobutanol

SI No	Patent No	Patent Title	Year	Reference
1	US 8,017,375 B2	Yeast Organism Producing Isobutanol At A High Yield	2011	[57]
2	US 9,303.225 B2	Method For The Production Of Isobutanol By Recombinant yeast	2016	[58]
3	US 9,284,612 B2	Fermentative Production Of Isobutanol Using Highly Active Ketol-AcdReductoisomerase Enzymes	2016	[59]
4	US 2010/0120105 A1	Carbon Pathway Optimized Production Hosts for the Production of Isobutanol	2010	[60]
5	US 8,373,012 B2	Renewable jet fuel blend stock from isobutanol	2013	[61]

Isobutanol production by native strain is reported in yeast *Saccharomyces cerevisiae* which uses valine biosynthesis pathway and Ehrlich pathway. However the isobutanol titers obtained by native strains are too low. Therefore metabolic engineering of host cells for improved isobutanol production has been studied. One of the major limitations of yeast cells is that the isobutanol pathway is compartmentalized to cytosol and mitochondria. Studies and reports suggest that confining the entire pathway either to cytosol or to mitochondria improves the isobutanol titer [6–8]. Experimental studies of overexpression of certain genes present in the pathway also suggest that isobutanol production is being improved. Availability of pyruvate is another problem that affect the production therefore by blocking competing pathway has also been studied for isobutanol production [9,10]. Beside yeast various microorganisms are also being studied for isobutanol production. Heterologous expression of genes studied in various organisms like *Escherichia coli, Bacillus subtilis, Corynebacterium glutamicum,* etc., showed improved isobutanol production [11].

Instead of using whole cell, cell free system has been used for isobutanol production. Isobutanol is produced *in vitro* by using enzymes, substrates, and cofactors. In case of cell free system substrate can be completely used for concerned product but in case of cellular approach substrate can be utilized by other competing pathway to produce by-products. Tolerance of bacteria cell to the product is another problem related to cellular approach but in cell free approach higher concentration of product does not affect the process. Productivity and yield is also higher in case of cell free system. Another advantage of cell free system is that it can operate at broad range of different conditions like pH, temperature etc [12].

## 2. Current isobutanol production strategies using microorganisms

### 2.1. Native strains

**Figure 2.**
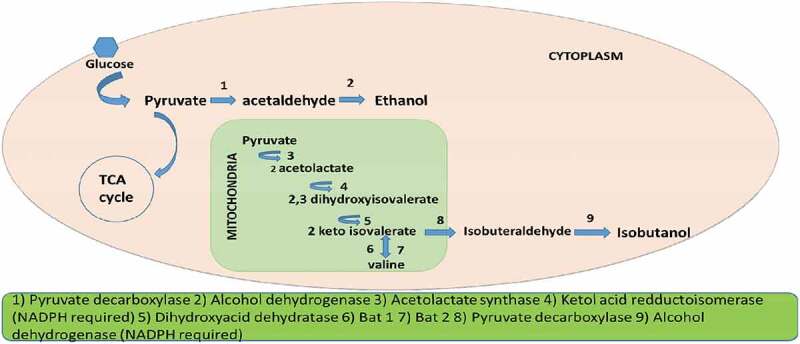
Isobutanol pathway in yeast

Researches on isobutanol production by wild type strains are less. The major reason for that are low productivity and also the limitation of genes responsible for isobutanol production in microorganisms. Earlier in 1990s there are few primary reports on trace amounts of isobutanol production detected as part of wine fermentation by few yeast species. The production range varies from 2 mg/L to 174 mg/L [13–15]. Later optimization of the *Saccharomyces cerevisiae* based on response methodology was performed which yield a productivity of 200 mg/L concentration of isobutanol [16]. In case of *S. cerevisiae* major drawback is that the pathway is distributed in to two compartments half in cytosol and half in mitochondria. Metabolic engineering approaches like over expression and exogenous gene expression were studied which will be discussing in later. In yeast isobutanol is synthesized through Ehrlich pathway by degradation of valine. Isobutanol pathway for yeast is depicted in Figure 2.

Tolerance of yeast species toward alcohol makes it a potential candidate for industrial purposes. Cell toxicity and tolerance toward isobutanol is one of major hindrance for production of isobutanol. Yeast is well studied for ethanol production due to its alcohol tolerance and is commercially used organism. Tolerance toward isobutanol is being documented which shows it is capable to grow in more than 2% [17]. This is the tolerance capacity of organism without any genetic alteration. Further modification of the genotype can have a more tolerant *S. cerevisiae*. Another added advantages of yeast is that no risk of phage contamination, which is common in bacterial fermentation.

Besides *S. cerevisiae,* other reported yeast strain for isobutanol includes *Pichia pastoris, Kluyveromyces lactis,* and *Magnusiomyces magnusii*. The wild strain of *P. pastoris* could produce only very low concentrations of isobutanol. Overexpression of endogenous amino acid biosynthetic pathway of *P. pastoris* led to an accumulation of 2.22 g/L of isobutanol [18]. *K. lactis* was studied to understand about the flavored volatile compounds produced. Isobutanol titer of 8.6 mg/L was detected from *K. lactis* during fermentation [19]. Recently multinuclear yeast *M. magnusii* was also reported for isobutanol production of 440 mg/L for a wild type strain. They also developed advanced transformation protocol in which strong constitutive promoter *TEF1* cloned and created the reporter system to test promoter strength. These methods also allowed to express heterologous *S. cerevisiae ILV2* gene coding for acetolactate synthase which led to advanced isobutanol production of 0.7 g/ L [20].

Bacterial isobutanol production by wild type strain is rare. Genes responsible for isobutanol production are absent in most of the bacteria. Ketoisovalerate decarboxylase is one such enzyme which is absent in bacteria.

This enzyme converts 2 ketoisovalerate to isobutyraldehyde which is the second last step of isobutanol production. *Lactococcus lactis* is a lactic acid bacterium which harbors Ketoisovalerate decarboxylase (KIVD) gene. In most genetic engineering approaches for bacterial isobutanol production this gene is introduced to bacteria from *L. lactis. L. lactis* is also being studied for isobutanol production since it contains all the genes. They used native *L. lactis, E. coli* DH5α which contains a KIVD gene from *L. lactis* and also combination of both. After fermentation the result showed positive isobutanol production for all three. Maximum production of 0.00173 g/mL was detected *for E. coli* DH5α. *L. lactis* showed production of 0.00001 g/mL. The co-culture reported the production of 0.00007 g/mL. This result confirmed *L. lactis* is a native isobutanol producer. The major disadvantage is the productivity and yield which is too low (Less than 0.2%) [21].

Isobutanol synthesis pathway is reported in *Klebsiella pneumonia* but this pathway is dormant in wild type strain. *K. pneumonia* is a well-known 2,3-butanediol producer. 2,3-butanediol and isobutanol is produced from pyruvate by condensation to yield α acetolactate. Inactivation of α acetolactate decarboxylase has been investigated which resulted in conversion of α-acetolactate toward valine pathway which finally leads to isobutanol synthesis. Further genetic modification was also done which increased the isobutanol concentration [22].

### 2.2. Genetic engineering approach for improved isobutanol production

#### 2.2.1. Bacterial genetics and approaches for isobutanol production

Isobutanol production by metabolic engineering of microorganisms has been well studied and shed a light into the various genes and its rate limiting factors that are associated with the production process. The common method adopted for bacteria is the introduction of last two step of Ehrlich pathway. The second last steps catalyze the conversion of ketoisovalerate to isobutyraldehyde by ketoisovalerate decarboxylase (KIVD). Most of bacteria lack this gene which is commonly found in plant, yeast, and fungi. *L. lactis* is the only commonly known organism which contains this KIVD enzyme. KIVD gene from *L. lactis* is broadly being studied for isobutanol production. The last step in Ehrlich pathway is the conversion of isobutyraldehyde to isobutanol aided by enzyme alcohol dehydrogenase (ADH). Alcohol dehydrogenase from different species has been studied to understand the most favorable one for isobutanol production which efficiently converts isobutyraldehyde to isobutanol. Besides introduction of last two genes various other modifications which include overexpression, inactivation of competent pathway, and genesis are also done to improve isobutanol production depending on the host studied. The improvement of process conditions also further improves the isobutanol yield [11].

Atsumi et al. were the first to carry out genetic engineering in *E. coli* strain for isobutanol production. Here he introduced last two steps of Ehrlich pathway into *E. coli*. He studied five different KIVDs, three from *S. cerevisiae* (Pdc6, Aro10, Thi3), KIVD from *L. lactis* and PDC from *Clostridium acetobutylicum*. The alcohol dehydrogenase enzyme was taken from *S. cerevisiae*. All the introduced genes were overexpressed to study the isobutanol production. The most efficient gene in *E. coli* was found to be KIVD from *L. lactis*. Beside the introduction of last two steps they try to increase keto acid flux by overexpression of ilvIHCD gene under control of PLlacO_1_ promoter. This led to a ≈ 5 fold increase as compared to gene without overexpression of ilvIHCD gene. Further isobutanol improvement was done by deleting gene that contributes by-product formation so that availability of pyruvate is increased. Instead of ilvIH of *E. coli* they introduced Als gene from *Bacillus subtilis*. The combined effect of all these manipulation lead to a final titer of 22 g/L of isobutanol production [23].

Aerobic isobutanol has been widely been studied since anaerobic production has an imbalance in cofactor utilization. In isobutanol production pathway two enzymes namely ketol acid reductoisomerase and alcohol dehydrogenase require nicotinamide dinucleotide phosphate (NADPH). Glycolysis produces only nictoniamidedinuleotide (NADH). In aerobic fermentation cell utilizes the NADPH produced by tricarboxylic acid cycle and pentose phosphate pathway. Bastion et al. have worked out two approaches to compete the cofactor imbalance. First approach uses the overexpression of pyridine nucleotide transhydrogenase PntAB to regenerate NADPH. Overexpression of transhydrogenase gene is one possible strategy to increase the availability of NADPH but the implementation of this in other microorganisms may not always shift the hydride ion in proper direction. Hence the second approach is found to be more effective. The second approach is shifting the NADPH-dependent pathway to NADH-dependent pathway. First they reduce the NADPH dependency partially which led to an increase of 2–3 fold as compared to NADPH dependent pathway. Finally by completely removing NADPH dependency they received isobutanol titer of 13.4 g/L which is 100% theoretical yield [24,25].

Similar studies were done by Aiqin et al., in their experiment they tackled the cofactor imbalance by overexpression of plasmid containing transhydrogenase gene and NAD kinase gene (pntAB and yfjB gene) and chromosomal modulation of transhydrogenase gene and NAD kinase gene. Plasmid overexpression showed only little effect on the production. The combinational chromosomal modulation of both the genes showed 50 and 30% increase in isobutanol titer and yield. Ultimately it was concluded from their study to have a positive effect on chromosomal modulation than using plasmid. The NADPH supply was increased by this method [26].

Another study related to this in 2019 Deb et al. used *E. coli*; here they addressed the disadvantages due to plasmid instability and antibiotic to maintain selection pressure. Other problem which they addressed is same as above which is cofactor imbalance. They constructed *E. coli* strain which has chromosomally integrated isobutanol pathway rather than plasmid insertion. The enzyme for this pathway was selected based on cofactor preference. In that study enzyme which prefers NADH was selected as a cofactor instead of NADPH. They also diverted the pyruvate flux toward isobutanol production by deleting the by-product formation [27].

From all above possible and effective solution for cofactor imbalance and to produce isobutanol titer include – chromosomal integration of pathway by selecting the appropriate cofactor and chromosomal overexpression of pntAB and NAD kinase for efficient NADPH production, shifting the cofactor from NADPH to NADH and deleting or inactivating the competing pathway to divert pyruvate flux toward isobutanol production.

Another metabolic engineering method by *E. coli* is adopting Entner–Doudoroff (ED) pathway. Glucose degradation uses normally Embden–Meyerhof (EM) pathway to produce isobutanol. However recently it is reported that Entner–Doudoroff pathway is being attempted for isobutanol production. ED pathway is notable in Gram negative bacteria, few Gram positive bacteria and archaea. In case of EM pathway which acquire energy from glucose uses 1 mol of glucose to produce 2 mol of pyruvate. In this process 2 ATP and 2 NADH is being produced. While in ED pathway 1 mol of glucose metabolized to 2 mol of pyruvate and produces 1 mol ATP, NADH and NADPH. ED pathway requires only few enzymes as compared to EM pathway. Isobutanol production from pyruvate requires 2 mol of NADPH. Therefore on that basis researcher constructed this ED pathway in *E. coli* due to redox balance. An active ED pathway constructed by suppressing the negative repressor of the pathway and genes of initial reaction of EM and pentose phosphate pathway (PPP). They also inactivated genes concerning organic acid. All together this improved isobutanol production. They obtained a final titer of 15 g/L of isobutanol [28,29].

The highest isobutanol production in *E. coli* was reported to be 50 g/L in 72 hrs and they have used constant product removal to tackle the toxicity of the product. The strain used was *E. coli* JCL 260 [30]. Various other attempts for *E. coli* isobutanol production include utilization of acetate to produce isobutanol. Advantages include no limiting factors as in case of glucose as carbon source [31]. Cellobionic acid was also attempted to use as sole carbon source by an engineered *E. coli* strain. The major genes responsible for growth on cellobionic acid are 6-phospho-beta-glucosidase (AscB) gene. Cellobionic acid broken to glucose 6 phosphate and gluconate further converted to isobutanol. The major drawback of the process is the cost of cellobionic acid is high. In order to reduce the cost waste containing cellobionic acid could be preferred [32].

Various other organisms also reported for isobutanol production by metabolic engineering approach which include *Corynebacterium glutamicum, Bacillus* species, *Shimwellia blattae, Cyanobacteria, Acetogenic* bacteria, *L. lactis, Geobacillus thermoglucosidasius, Enterobacter aerogenes, Clostridium ljungdahlii, Zymomonas mobilis, Ralstonia eutropha, Clostridium cellulolyticum, Pseudomonas Putida*. Methodology is similar as that which is adopted in *E. Coli* in most of the reports.

*Corynebacterium glutamicum* is a gram positive rod-shaped non-spore forming bacteria. The application of this organism in industries has widely improved in recent years due to its tolerance and more knowledge on metabolic engineering approaches. The cellular machinery of *C. glutamicum* is ample for amino acid production further which aid alcohol production. Extensive understanding of the genetic level modification opened up its application in divergent industrial process. Isobutanol production is being studied by various scientists using *C. glutamicum*. Initial studies of isobutanol production by *C. glutamicum* harbors 4.9 g/L **[**33**]**. Further researcher has gradually improved the production titer in *C. glutamicum*. Different metabolic engineering methodology to improve yield include 1) Inactivated L-lactate and malate dehydrogenase, inserted KIVD gene from *L. lactis*, ADH2 from S. cerevisiae and pntAB from *E. coli*. Further chromosomally integrated adhA was used for improved production. Final isobutanol production of 175 mM was obtained **[**34**]**. 2) Introduction of ED pathway which enhances glucose consumption and productivity. Here they used enzymes which prefer NADH rather than NADPH. They also inactivated succinate by production. Overall isobutanol production of 280 Mm was obtained in 24 hrs **[**35**]**. 3) Disruption of ppc gene, encoding phosphoenolpyruvate carboxylase, NADH preferred acetohydroxylacidisomeroreductase (AHAIR) was selected instead of NADPH dependent, overexpressing select glycolytic gene. Beside all this they continuously extracted isobutanol based on oleyl alcohol method. Final volumetric production of 981 mM was obtained from *C. glutamicum* **[**36**]** 4) *C. glutamicum* engineered to use hemicellulose fraction. Integrated genes required for D-xylose and L-arabinose utilization. Isobutanol of 7.2 mM was obtained **[**37**].**

*Bacillus subtilis* is also an appropriate host for isobutanol production due to its tolerance and extensive substrate utilization ability. Isobutanol biosynthesis occurs by 2 ketoisovalerate pathway with addition of heterologous ketoacid decarboxylase and alcohol dehydrogenase. Initial study on *B. subtilis* produced 0.6 g/L [38]. Later isobutanol production was increased by over expressing acetolactate synthase. Acetolactate synthase overexpression showed isobutanol production of 2.62 g/L [39].

**Figure 3.**
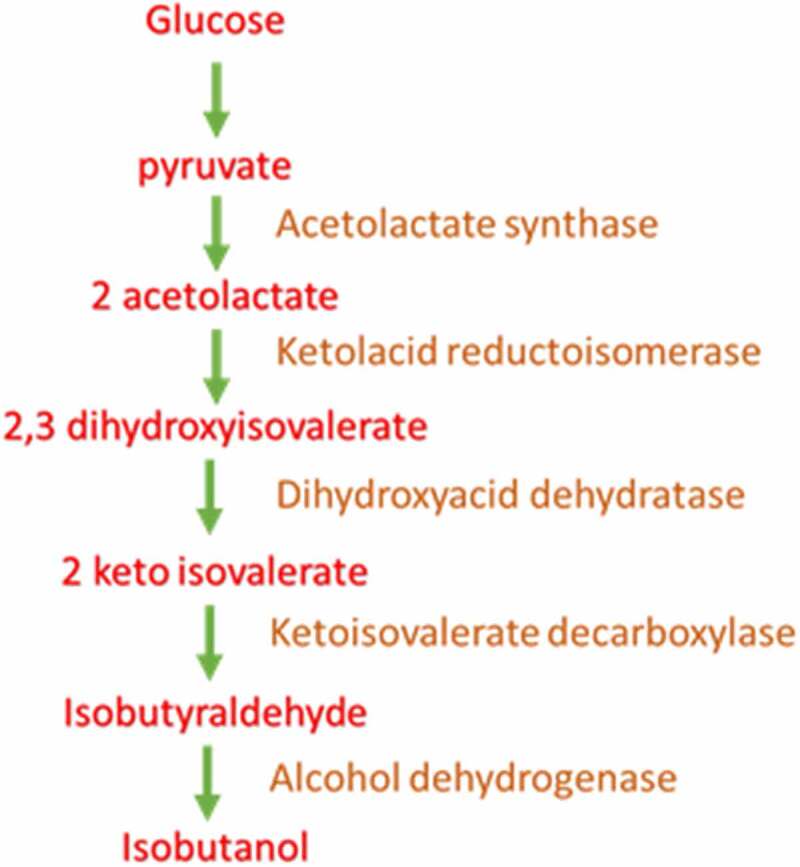
Bacterial pathway for isobutanol synthesis

All other bacterial reports are also similar which include, *Shimwellia blattae, Cyanobacteria, Acetogenic bacteria, L. lactis, Geobacillus thermoglucosidasius, Enterobacter aerogenes, Clostridium ljungdahlii, Zymomonas mobilis, Ralstonia eutropha, Clostridium cellulolyticum, Pseudomonas putida*. Mostly last two steps were introduced heterologously that is KIVD and ADH enzyme. Further cofactor is being balanced by engineering approach. Finally media optimization is also been done [40–48]. All together they produce isobutanol from bacteria. The bacterial isobutanol pathway is depicted in Figure 3.

#### 2.2.2. Yeast genetics for isobutanol production

In microbial isobutanol production major hindrance is they have to introduce heterologously the genes for isobutanol production. Most bacteria lack ketoisovalerate decarboxylase (KIVD) which aids the conversion of 2 ketoisovalerate to isobutyraldehyde. The yeast has a well-established isobutanol pathway. In addition yeast utilizes efficiently lignocellulose biomass and also can with stand harsh conditions. Yeast especially *S. cerevisiae* will produce higher alcohols like isobutanol in small amount through valine biosynthesis and Ehrlich pathway.

In *S. cerevisiae* isobutanol pathway is distributed in mitochondria and cytosol. Initial steps in isobutanol production which include valine biosynthesis occur in mitochondria and the rest so called Ehrlich pathway is confined to cytosol. Pyruvate produced through glycolysis are transferred into the mitochondria through the mitochondrial pyruvate complex [49].The pyruvate get converted to 2 acetolactate by acetolactate synthase enzyme (ILV2). Further the acetolactate is reduced to 2,3-dihydroxyisovalerate by ketol acid reductoisomerase which is NADPH dependent. 2,3-dihydroxyisovalerate is then dehydrated to form 2 ketoisovalerate which is catalyzed by dihydroxyacid dehydratase. Final step of valine biosynthesis from 2-ketoisovalerate aided by BAT 1 enzyme also occur in mitochondria.

The proceeding reaction of Ehrlich pathway for isobutanol occurs in cytosol. BAT 2 present in cytosol by its transaminase activity convert valine to 2 ketoisovalerate (KIV). 2-ketoisovalerate in cytosol gets converted to isobutyraldehyde by pyruvate decarboxylase. Genes responsible for this conversion include PDC1, PDC5, PDC6, ARO10, THI3. Formed isobutyraldehyde is reduced to isobutanol by alcohol dehydrogenase enzyme which require NADPH as cofactor [50].

Different techniques were evaluated by scientists across the globe to understand the effect of isobutanol production by yeast. First three gene starting from pyruvate (ILV2, ILV5, ILV3) and BAT2 were over expressed. Overexpression of these three genes increased isobutanol production from 0.16 to 0.97 mg/g of glucose [51]. In another report they have over expressed the entire gene in pathway which includes ILV2, ILV5, ILV3, ARO 10 and ADH2. Besides this they also deleted ALD6 AND BAT1 gene. They obtained isobutanol production of 376 mg/L [40].

Rather than overexpressing another approach applied include translocation of isobutanol biosynthesis pathway entirely to cytosol. In addition to the translocation they also overexpressed KIVD, ARO10, and alcohol dehydrogenase .Final yield of 0.63 g/L was obtained [7]. Instead of glucose utilization *S. cerevisiae* was engineered to utilize xylose. For efficient utilization of xylose by *S. cerevisiae* xylose isomerase, transaldolase and xylulokinase genes were overexpressed. In this study over expression of KIVD, ARO10 and ADH2 were carried out in which all the genes for isobutanol production were localized cytosolically. Final titer of 1.36 mg/L was obtained [41]. Instead of transferring entire biosynthetic pathway to cytosol, another strategy includes mitochondrially targeting the isobutanol pathway. In this study they coupled xylose consumption with targeting the isobutanol pathway to mitochondria. Further optimization of culture media produced 2.6 g/L of isobutanol [42].

Mutagenesis is another method adopted for increasing isobutanol. Ethyl methyl sulfonate mutageneses with adaptive laboratory evolution for improving tolerance have done. In addition to this they also overexpressed ILV3, ILV2, ILV5,and ARO which resulted in improved production of 4 g/L [43]. As we discussed above in case of bacteria regarding ED pathway a similar approach for yeast has also attempted to increase production. Phosphoenol pyruvate carboxylase (PPC) and ED pathway were expressed heterologously in *S. Cerevisiae* to understand their role in isobutanol production. Heterologous expression of PPC improved the NADPH supply which is needed for isobutanol synthesis and also thus improved its production. Whereas heterologously expressed enzyme in ED pathway has no effect on isobutanol synthesis but have a role in improving the growth rate [44].

Along with mitochondrial localization of pathway improving the mitochondrial pool of pyruvate availability have a positive result in isobutanol production. Mitochondrial pyruvate carriers (MPC) which include mpc1, mpc2, and mpc3 were overexpressed in various combinations. Overexpression of mpc1 and mpc 3 improved isobutanol production. Improvement of 22 fold with an isobutanol titer of 330.9 mg/L from 20 g glucose was obtained [45].

These are the different approaches used in yeast for improving isobutanol production. In most of the studies in yeast over expression of genes in isobutanol pathway along with localization of the pathway either to mitochondria or toward cytosol is attempted. In most of the cases they also block the competing pathway to redirect the entire pyruvate flux toward isobutanol synthesis. Many commercial companies like Gevo located in Douglas County, Colorado, use yeast for isobutanol production.They claim to have theoretical yield of 97.5%. They use crab positive and crab negative yeast with added product removal system. They engineered strain by overexpressing isobutanol pathway and also by deleting PDC gene. Yeast is a potential host for isobutanol production. Production profile of isobutanol from different microorganisms is depicted in Table 2.

**Table 2.** Isobutanol production profile of different microorganisms

			Concentration of isobutanol		
Host strain	Carbon source	Genes over expressed	(g/L)	Pathways involved	Reference
*Bacillus subtilis*	glucose	kivd,adh2	0.607	Ketoacid pathway	[38]
*Bacillus subtilis*	glucose	alsS,ilvCD,kivd,adh2	2.62	Ketoacid pathway	[39]
*Corynebacterium glutamicum*	glucose	ΔpckA,ilvBNCD,adhA,kivd	˜12.8	Entner–Doudoroff pathway and ketoacid pathway	[35]
*Corynebacterium glutamicum*	glucose	Δppc, ilvBNCD, kivd, adh	˜45	Ketoacid pathway	[36]
*Escherichia coli*	glucose	alsS,ilvCD,kivd,adhA	22	Ketoacid pathway	[23]
*Escherichia coli*	glucose	alsS,ilvCD,kivd,adhA	50	Ketoacid pathway	[30]
*Shimwellia blattae*	glucose	pntAB,adh	5.98	Keto acid pathway	[62]
*Enterobacter aerogenes*	glucose	ΔldhA, ΔbudA, ΔpflB,kivd,adhA,ilvCD,budB	4.3	Keto acid pathway	[63]
*Saccharomyces cerevisiae*	xylose	xyl,ILV2,ILV3,ILV5, kivd, adh	2.6	Pentose phosphate pathway and ketoacid pathway	[42]
*Saccharomyces cerevisiae*	glucose	ILV2,ILV3,ILV5,KIVD,AADH	0.143	Keto acid pathway	[64]

**Figure 4.**
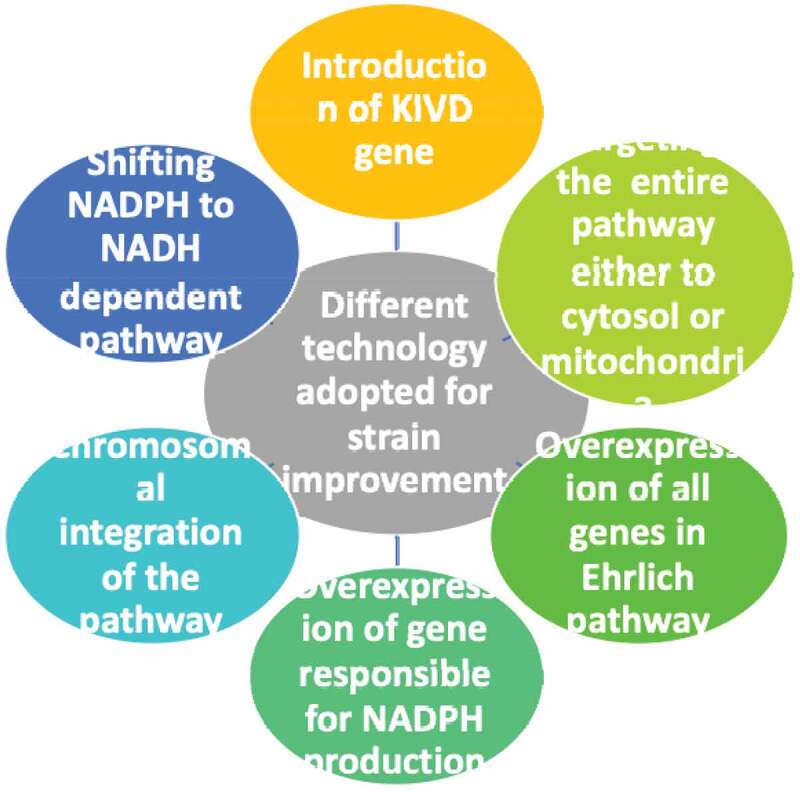
Different strain improvement methods used for isobutanol production

Different strain improvement strategies used for bacteria and yeast cell are shown in Figure 4

### 2.3. Cell free approach for isobutanol production

**Figure 5.**
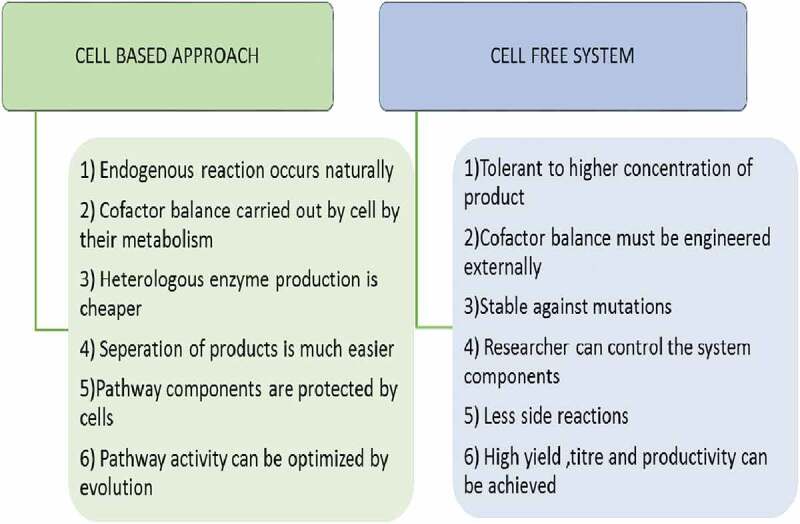
Advantages of whole cell and cell free system for production of value-added products

Besides the whole cell method for isobutanol production, an alternative strategy includes cell free bio-production system. In cell free system, substrate, enzymes and cofactors are added together and is converted into corresponding product. This originates from so called biocatalyst field. Comparison of whole cell based and cell free approach is shown in Figure 5. In one of the cell free experiment they immobilized two enzyme namely kdcA and ADH (from *S. Cerevisiae*) on methacrylate resin. Another enzyme FDH required for NADH recycle is added to solution. Substrate used was ketoisovalerate. Product conversion of 55% was achieved by this method [46].

Similar type of experiment with cell free system was carried out by another group of researcher in which they received a yield of 95% and isobutanol titer of 275 g/L within 5 days. This was the higher titer reported till date. Here they used a bioreactor with the enzymes that are tolerant to isobutanol. The production rate is high but the major complication with cell free approach is the cost of enzyme and cofactor. Once we can replace it with cheaper cofactor and enzymes this process open up a broad area for commercial production of biofuel using cell free system [47].

### 2.4. Lignocellulosic biomass as substrate for isobutanol production

Earlier biofuel industries focus on the production based on first generation biofuel. This will lead to a competition with animal feed and food industries. To avoid this later lignocellulosic biomass like plant and crop waste was selected as feedstock for biofuel production. Lignocellulosic biomass is one of the most available renewable wastes that can be utilized. Lignocellulosic biomass is made up of polysaccharides (cellulose and hemicellulose) and lignin. Due to its complex structure lignocellulose material should undergo pretreatment using chemical so as to remove lignin and followed by hydrolysis step in which enzyme added to break cellulose and hemicellulose into sugar monomer. Final fermentation using this result in biofuel production [48].

Instead of separate hydrolysis and fermentation, simultaneous saccharification and fermentation (SSF) is a process which combines hydrolysis and fermentation via single step. Most industries prefer simultaneous saccharification and fermentation due to lower cost, less inhibitory compounds and also low risk of contamination. Concern regarding simultaneous saccharification and fermentation include optimum pH since the condition might be different for hydrolysis and fermentation. Therefore there is a necessity to find an equilibrium where both the process works efficiently [52].

Nowadays consolidated bioprocessing(CBP) is being so as to reduce the cost of pretreatment and hydrolysis step. Consolidated bioprocessing uses single step approach in which enzyme production, biomass hydrolysis, and fermentation are accomplished in a single process step by lignocellulolytic microorganisms.

**Figure 6.**
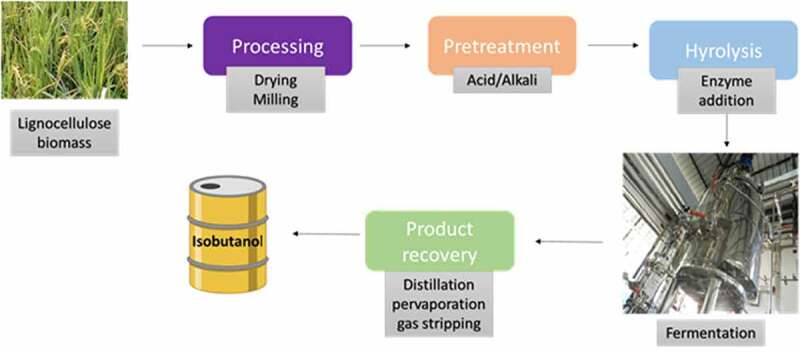
Overall isobutanol production from Lignocellulosic biomass

There are only few reports on isobutanol production using lignocellulosic biomass. There is a large scope for isobutanol using lignocellulose material. Consolidated bioprocessing was experimented using *Clostridium thermocellum* for isobutanol production. Metabolically engineered *C. thermocellum* able to utilize cellulose and can produce isobutanol. *C. thermocellum* is a well known cellulose utilizer which is used for CBP, therefore they engineered the genes responsible for isobutanol production and the plasmid was chromosomally integrated into *C. thermocellum*. Final titer of 5.4 g/L isobutanol obtained from cellulose with 75 hrs which correspond to theoretical yield of 41% [53]. Overall figure showing isobutanol production from lignocellulosic biomass is shown in Figure 6

### 2.5. Downstream processes for isobutanol separation

In situ removal of n-butanol has been reported but for isobutanol there are only lower reports as compared to n-butanol. For n-butanol various techniques like gas stripping, pervaporation, and adsorption have been evaluated. Isobutanol due to its lower concentration in fermentation media, energy consumption is high for its separation process [54].

One method of isobutanol purification and removal include rectification. Isobutanol containing fermentation broth is passed through different rectification tower. Finally the isobutanol with little remaining water is separated by using a cooler and decanter. The immiscible isobutanol-rich phase is refluxed to isobutanol stripper and is thus separated [54].

Pervaporation is a process in which compounds can be separated based on potential difference across the membrane. Based on the affinity of membrane, molecules having affinity toward membrane will adsorb and diffuses while the others with low affinity is being retained. For isobutanol separation using this method polydimethylsiloxane (PDMS) membrane is used to reduce the toxicity. Fermentation coupled with pervaporation was done using *Enterobacter aerogens*. They used PDMS membrane casted on polyvinylidene fluoride for pervaporation. The condensate contained 55–226 g/m^2^h of isobutanol [54].

Gas stripping is also an effective recovery strategy. In gas stripping feed is injected toward stripping jar and hot gas is supplied. Volatile difference of the compound is used to separate the solvents from fermentation media. For isobutanol separation, coupled gas stripping and fermentation carried out which produced 50 g/L of isobutanol [30]. Isobutanol yield is double as compared to n-butanol with same condition.

Salting out extraction is another methodology in which organic solvent and salt is used for separation. Salting out is already known technique for n butanol. In salt out extraction requires low amount of salt and is more efficient separation. Different salts were tried out for isobutanol separation and potassium pyrophosphate proved to be the best one. Among different extractants 2-ethyl 1 hexanol served as best host. Isobutanol recovery of 100% was achieved by this method [55].

**Figure 7.**
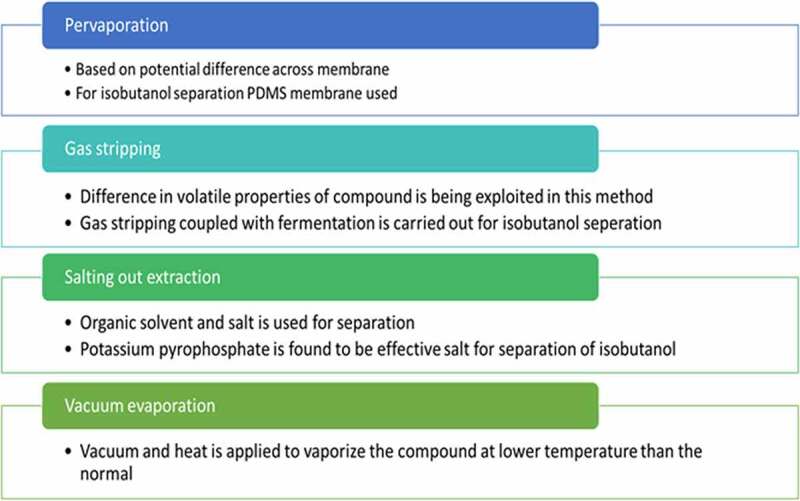
Different downstream process used for isobutanol separation

Vacuum evaporation is also a separation technique used commonly. This process uses vacuum and heat to vaporize the compound at a reduced pressure at lower boiling point. This method has been experimented for separation of isobutanol. Isobutanol of 15 g/L occurred at ≤ 34.3° [56]. Various other methods adopted for separation of isobutanol include adsorption, solvent extraction etc. By simple distillation also it is possible to separate isobutanol. Since isobutanol solubility in water is low separation is much easier [54]. Different isobutanol separation techniques with its discussion have been shown in Figure 7

## 3. Conclusions and future perspectives

Recent advances and studies on isobutanol production in microorganisms open up a platform for successful production of isobutanol. Bacterial and yeast strains have been studied for isobutanol production. Due to lack of a proper pathway and also the genes responsible for isobutanol production, there is the major hindrance in bacterial cell to produce this alcohol. This problem was addressed by genetic engineering approach in which isobutanol pathway was heterologously introduced into bacterial cell. NADPH availability is another factor responsible for lower production which was addressed by using the enzyme which requires NADH as cofactor instead of NADPH. Another strategy to address this issue includes improving NADPH production by overexpressing pyridine nucleotide transhydrogenase and also chromosomal integration of the genes.

Yeast cells have isobutanol production pathway which is confined to mitochondria and cytosol. Strategies used to improve isobutanol production include overexpression of genes involved in this pathway, localization of the pathway either to cytosol or mitochondria, blocking the competitive pathway to obstruct the by-product formation and also improved NADPH availability.

In yeast and bacterial cell major limitation include tolerance of microorganism to the product due to the product toxicity. This can be addressed both by using a tolerant strain and through genetic engineering approach the tolerance could be improved. Improving the microbial phenotype by adopting methods like adaptive evolution could open up future directions to improve the production of isobutanol by reducing the product toxicity. Another approach is the integrated removal of the product as and when produced.

Addition to all these, for an environmental friendly and sustainable production of isobutanol, there is a need to develop efficient methods for the utilization of cheap raw materials such as lignocellulose biomass or other industrial and municipal wastes. There are only a few studies are reported for microbial isobutanol production in this direction. Hence there exists a wide scope for research in this area.

Isobutanol production urged importance few years ago, but the commercial production of isobutanol is low mainly due to the low productivity issues due to various reasons like cell toxicity, scarcity of NADPH availability which has been addressed in various studies as mentioned above. Based on the available knowledge, engineering of an effective strain and utilization of lignocellulosic waste as substrate helps to open up a wide possibility for commercial production of isobutanol.
